# FGFR1, a novel biomarker for metastatic castration-resistant prostate cancer?

**DOI:** 10.18632/oncotarget.27957

**Published:** 2021-05-25

**Authors:** Leandro H. Gallo

**Keywords:** prostate cancer, metastasis, cancer stem cells, castration-resistant, tyrosine kinase inhibitors

***News on:** Characterization of FGFR signaling in prostate cancer stem cells and inhibition via TKI treatment by Ko et al. Oncotarget. 2021; 12:22–36. https://doi.org/10.18632/oncotarget.27859. [PubMed]*

Prostate cancer is the second most common cause of cancer-related death among men in the United States [1]. In a seminal paper eighty years ago, Huggins and Hodges demonstrated that prostate tumors and metastatic disease are sensitive to the presence or absence of androgenic hormones [2]. Remarkable milestones in terms of treatments have been achieved clinically, in which early diagnosis can lead to curative treatments prior to disseminated metastasis. Unfortunately, most patients, some of whom have undergone hormone deprivation therapy, will present with recurrent castration-resistant disease or metastatic tumors with high incidence and short survival [3].

In 2013, researchers at the University of California, San Diego identified a subpopulation of androgen-unresponsive cells in early prostate tumors derived from surgical specimens of prostatectomy cases. These cells express stem cell markers such as CD44, CD133, ALDH1, TERT, Oct-4, Sox-2 and Nanog, exhibit anchorage-independent spheroid growth in matrigel, are poorly differentiated, and present a widely disseminated phenotype when surgically engrafted *in vivo* [4]. Interestingly, these cells exhibit lower expression of prostate specific antigen (PSA), a powerful observation worth of mentioning briefly towards the end of this commentary. Later on, the same group showed that some human prostate cancer cells exhibit a stem cell-like phenotype, suggesting that prostate cancer is a stem cell disease that arises from early cancer-associated reprogramming mechanisms [5]. However, the identity of proteins with oncogenic properties that modulate such cancer-associated reprogramming mechanism of androgen-unresponsive cells remained elusive.

**Figure 1:**
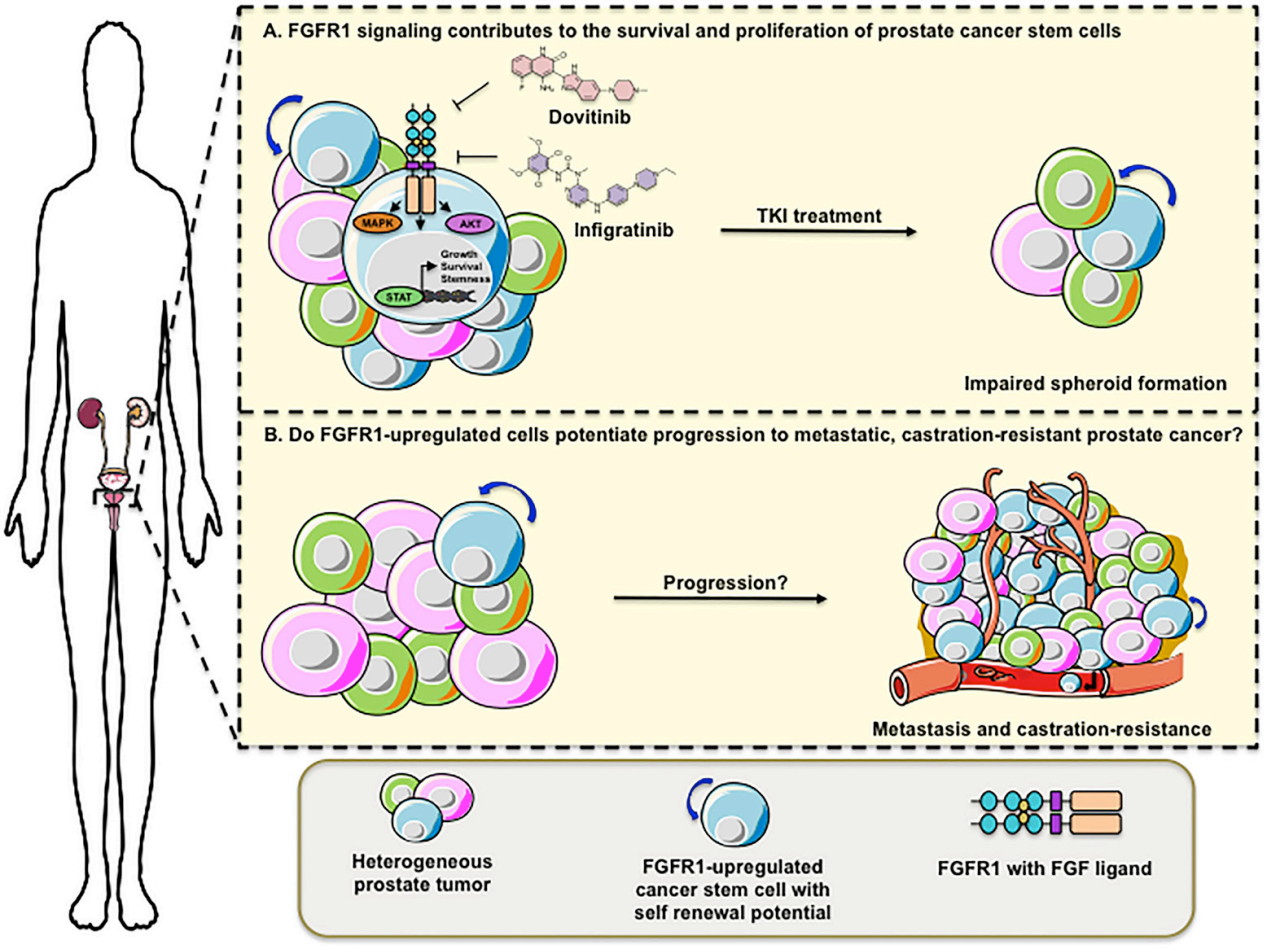
FGFR1 contributes to the stemness of prostate cancer cells with tumor initiating potential. (**A**) Tumors usually exhibit a heterogeneous phenotype with subpopulations of cells at different clonal stages as illustrated by cells colored in green, magenta, and blue. Upregulation of FGFR1 signaling (shown in blue colored cells) leads to the activation of oncogenic MAPK, AKT, and STATs, culminating in cellular survival, proliferation, and expression of stem cell markers. Treatment with tyrosine kinase inhibitors (TKI), Dovitinib and Infigratinib (BJG398), impairs spheroid formation of prostate cancer stem cells. (**B**) It remains to be investigated whether FGFR1-upregulated cancer stem cells potentiate progression to metastatic and castration-resistant prostate cancer.

Tumors usually exhibit a heterogeneous phenotype in which subpopulations of cells are present at different clonal stages. Recently, Ko J, et al. published a paper that attempts to unravel the clue about the aggressive nature of androgen-unresponsive cells in prostate cancer [6]. They reported that these cells depend on fibroblast growth factor receptor (FGFR), specifically FGFR1, for spheroid formation and activation of oncogenic pathways involving MAPK, STATs, and AKT signaling. In addition, the authors showed that pharmacological treatments of PC3 and DU145 prostate cancer cell lines with Infigratinib (BJG398) and Dovitinib impaired spheroid growth *in vitro*. A recently identified cell line - iPS87 - with pluripotent stem cell properties published by the same group [7], showed pharmacological response to Infigratinib and Dovitinib treatments in the nanomolar range, which is clinically significant. iPS87 cells treated with TKIs exhibited lower expression of FGFR1 and stem-cell markers, in addition to decreased survival and proliferation of the spheroids (Figure 1A).

Altogether, this body of work suggests that FGFR activation is critical for the proliferation and maintenance of stemness of different subtypes of prostate cancer cells. It remains to be investigated whether FGFR1-upregulated cells contribute to the progression of benign lesions to metastatic, castration-resistant prostate tumors (Figure 1B). Furthermore, it remains to be investigated whether FGFR1-upregulated cancer stem cells modulate the plasticity of prostate tumors, contributing to the transformation of adenocarcinoma into a highly aggressive neuroendocrine phenotype. For instance, patients with castration-resistant prostate cancer are eligible to receive hormone therapies like abiraterone (Zytiga, by Janssen) and enzalutamide (Xtandi, by Astellas Pharma), but most will develop drug resistance with unknown mechanisms. Men initially diagnosed with adenocarcinoma of the prostate have developed a different subset of prostate cancer termed treatment-emergent small-cell neuroendocrine prostate cancer (t-SCNC) after abiraterone and enzalutamide treatments [8]. Interestingly, this subset of prostate cancer exhibits low/absent expression of PSA [9]; this is reminiscent of prior observations by these researchers in their prostate tumor cohorts in which cultured prostate cancer cells and orthotopically-induced *in vivo* cancers lacked PSA expression [4].

First-generation TKI inhibitors, such as ponatinib, dovitinib, lucitanib, and anlotinib, exhibit a multi-target inhibition profile for VEGFR1-3, KIT, RET, including FGFR’s. This led to the development of novel agents with fine-tuned specificity for FGFR’s, some of which are currently being tested in clinical trials. For instance, erdafitinib, a reversible FGFR1-4 inhibitor [10], is being tested in patients with castration-resistant prostate cancer [NCT04754425], and in combination with abiraterone or enzalutamide in double-negative (AR-null/NE-null) prostate cancer [NCT03999515]. Double-negative prostate cancer has been previously attributed to FGFR/MAPK signaling [11]. Additional specific FGFR1-4 inhibitors are currently being tested, such as AZD4547 in refractory solid and hematological malignancies [NCT04439240], infigratinib as first-line therapy for FGFR2 translocation-positive cholangiocarcinoma [NCT03773302], pemigatinib in urothelial cancer [NCT04294277] and tumors with FGFR mutations and translocations [NCT04003623], rogaratinib in non-small cell lung cancer that overexpresses *FGFR1-3* transcripts [NCT03762122], and futibatinib alone or in combination with fulvestrant in patients with advanced/metastatic FGFR1- or FGFR2-amplified breast cancer [NCT04024436]. Depending on the outcome of these studies, we will have an assessment of the possibility that FGFR signaling contributes to the survival and proliferation of a subpopulation of stem cells with cancer initiating properties.
